# Designing the Crops for the Future; The CropBooster Program

**DOI:** 10.3390/biology10070690

**Published:** 2021-07-20

**Authors:** Jeremy Harbinson, Martin A. J. Parry, Jess Davies, Norbert Rolland, Francesco Loreto, Ralf Wilhelm, Karin Metzlaff, René Klein Lankhorst

**Affiliations:** 1 Laboratory of Biophysics, Department of Agrotechnology and Food Sciences, Wageningen University & Research, 6700 HB Wageningen, The Netherlands; jeremy.harbinson@wur.nl; 2 Lancaster Environment Centre, Lancaster University, Lancaster LA1 4YQ, UK; m.parry@lancaster.ac.uk (M.A.J.P.); jess.davies@lancaster.ac.uk (J.D.); 3 Centre for Global Eco-Innovation, Lancaster University, Lancaster LA1 4YQ, UK; 4 Laboratoire de Physiologie Cellulaire & Végétale, University Grenoble Alpes, CNRS, INRAE, CEA, 38 000 Grenoble, France; norbert.rolland@inrae.fr; 5 Department of Biology, Agriculture and Food Sciences, National Research Council (CNR), 00185 Rome, Italy; francesco.loreto@unina.it; 6 Department of Biology, University of Naples Federico II, 80138 Naples, Italy; 7 Institute for Biosafety in Plant Biotechnology, Julius Kühn-Institut, 06484 Quedlinburg, Germany; ralf.wilhelm@julius-kuehn.de; 8 European Plant Science Organisation (EPSO), 1000 Brussels, Belgium; Karin.Metzlaff@epsomail.org; 9 Wageningen Plant Research, Wageningen University & Research, 6708 PB Wageningen, The Netherlands

**Keywords:** food supply, climate change, crop yield, sustainability, resource use efficiency, photosynthesis, biodiversity, CO_2_, bioeconomy, breeding

## Abstract

**Simple Summary:**

Our climate is changing and the world population is growing to an estimated 10 billion people by 2050. This may cause serious problems in global food supply, protection of the environment and safeguarding Earth’s biodiversity. To face these challenges, agriculture will have to adapt and a key element in this will be the development of “future-proof” crops. These crops will not only have to be high-yielding, but also should be able to withstand future climate conditions and will have to make very efficient use of scarce resources such as water, phosphorus and minerals. Future crops should not only sustainably give access to sufficient, nutritious, and diverse food to a worldwide growing population, but also support the circular bio-based economy and contribute to a lower atmospheric CO_2_ concentration to counteract global warming. Future-proofing our crops is an urgent issue and a challenging goal that only can be realized by large-scale, international research cooperation. We call for international action and propose a pan-European research and innovation initiative, the CropBooster Program, to mobilize the European plant research community and all interested actors in agri-food research and innovation to face the challenge.

**Abstract:**

The realization of the full objectives of international policies targeting global food security and climate change mitigation, including the United Nation’s Sustainable Development Goals, the Paris Climate Agreement COP21 and the European Green Deal, requires that we (i) sustainably increase the yield, nutritional quality and biodiversity of major crop species, (ii) select climate-ready crops that are adapted to future weather dynamic and (iii) increase the resource use efficiency of crops for sustainably preserving natural resources. Ultimately, the grand challenge to be met by agriculture is to sustainably provide access to sufficient, nutritious and diverse food to a worldwide growing population, and to support the circular bio-based economy. Future-proofing our crops is an urgent issue and a challenging goal, involving a diversity of crop species in differing agricultural regimes and under multiple environmental drivers, providing versatile crop-breeding solutions within wider socio-economic-ecological systems. This goal can only be realized by a large-scale, international research cooperation. We call for international action and propose a pan-European research initiative, the CropBooster Program, to mobilize the European plant research community and interconnect it with the interdisciplinary expertise necessary to face the challenge.

## 1. A Need for Change

Plants, including algae and cyanobacteria, are photoautotrophs, taking simple inorganic substrates, such as CO_2_, and using energy from sunlight to produce energy-rich organic molecules. These photoautotrophs feed the biosphere and mankind is no exception. Human civilization has progressed using plants (including algae and cyanobacteria) as the sole primary source of energy organic molecules. Plants, directly or indirectly, delivered all our food and in addition underpinned much of our technology, providing building materials, fibers for clothing, feed for the production of farmed animals and fish, heat to warm houses and to prepare food, as well as the energy and some of the raw materials needed for basic manufacturing. Society largely relied on plants until the 18th century, when the exploitation of fossil fuels sparked the Industrial Revolution. Although fossil fuels are also derived from plants and other biological materials, the large-scale use of coal, gas and oil hallmarked the advent of a new economy: the fossil economy. This economy depended on the combustion of relatively abundant fossil fuels to drive the expansion of the heat-based manufacturing processes that characterized the industrial revolution.

As a result of the industrial revolution, fossil carbon became our main energy source and feedstock and the importance of plant products diminished. The unprecedented success of the new economy led to increased welfare in society that manifested, for instance, by an increased food availability both in quantity and quality, improved hygiene and advanced medical care. The fossil economy provided the machines needed to increase agricultural productivity, the fuel to drive the machines, the chemical processes to produce fertilizers and the manufacturing of pesticides. The increasing wealth generated by the fossil economy led to better education, in turn supporting the acceleration in the technological progress of agriculture. This improvement in agricultural productivity made the continued exponential growth of the human population possible.

However, the rise of human populations, global per capita consumption and dietary demands, combined with the need to address malnutrition and inequalities in many regions, is increasing the strain on our agricultural systems and our Earth’s ecosystem. Resources are also running out and some of the most common natural resources, such as fresh water [1], soils [2] and biodiversity [3] among natural and cultivated plants, are important for agriculture. Society’s dependence on fossil fuels has caused atmospheric CO_2_ to rise to dangerous levels, triggering global climate warming and change. Current agriculture also leads to the production of other greenhouse gases such as nitrous oxide and methane. Simultaneously, the increasing demand for food and feed has caused large-scale pressure on forests. Deforestation itself leads to loss of CO_2_ sinks, releases additional CO_2_ and causes land degradation and a loss of biodiversity and soil fertility [4].

In order to mitigate, halt or even reverse the negative effects of the fossil economy, society will have to progress towards a post-fossil society driven by more sustainable biological processes and transition to more sustainable technologies. In such a “bio-society”, plants once again become the primary source of all our organic materials, fibers, food and feed, and also contribute to clean fuel and energy demands, generated without the net emission of CO_2_.

**Figure 1:**
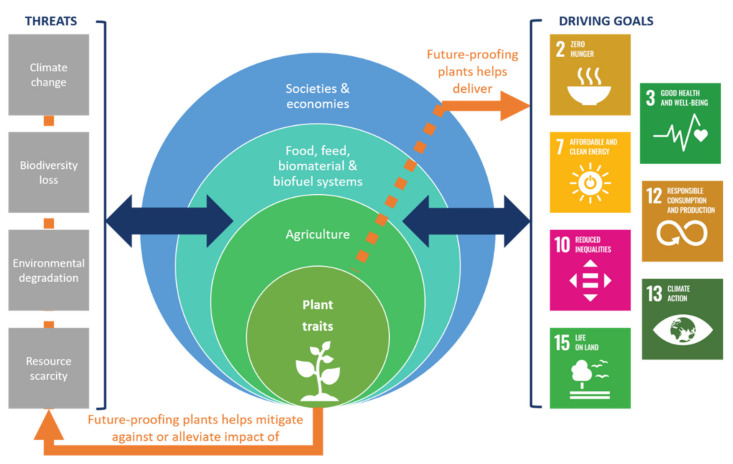
Plant traits are a key part of our agricultural systems, which underpin food, feed biomaterial and biofuel systems and in turn form the foundation of societies and economies. Our current agricultural and food/feed/fiber/fuel systems are both driving and exposed to a number of key threats that endanger their future. Likewise, the need to meet the sustainable development goals creates rising demands on our agri-food/feed/fiber/fuel systems to produce more and do so more sustainably. Plant trait innovation provides a means for future-proofing plants against the threats, and helping future-proof agriculture such that it can help deliver the SDGs.

Urgency is essential, as warnings increasingly suggest that it will soon be too late to reverse the adverse effects of global warming [5] and unsustainable use of resources [6]. An increasing number of calls for action are being made by developing international policies addressing global food and nutritional security, protection and use of biodiversity, increasing sustainability and resource use efficiency, climate change mitigation and adaptation [7]. Meeting the ambitions and commitments of United Nation’s Sustainable Development Goals (SDGs), the Paris Climate Agreement COP21 and the European Green Deal will be made easier if agriculture can once again help in meeting our primary needs (Figure 1).

Accomplishing this, however, is a daunting task. Sustainably meeting the health needs of all people by 2050 means we need to close yield gaps and increase global crop yields by 70–110% [8,9] whilst diversifying what we grow, radically improving nutrient and water use efficiency and rapidly moving agriculture away from a greenhouse gas emitter to a carbon sink [10]. Furthermore, the realization of the circular bio-based economy might require an additional 30% crop yield increase [11], which brings the total required global crop yield increase by 2050 to 100–140%. Enhancing production should also be systematically associated with quality of the food and food harvest stability, the correct parameter to consider being increased nutrient yield and quality per land use. Indeed, hidden hunger (1.8 billion people) is affecting twice the number of people suffering from caloric hunger [10].

In addition, a rise in crop productivity has to be accomplished in a sustainable manner without compromising biodiversity or negatively impacting natural resources and the environment. This implies that agricultural lands may not grow endlessly and must not outcompete services by natural vegetation. In fact, it is a prudent estimate that agricultural land area will even decrease in the coming decades [12,13]. Sustainable use of biodiversity can make the difference in this problematic landscape. Selecting plants that improve resource (water, mineral nutrients, soil) use efficiency, or improve the performance of under-utilized crop species and varieties, thereby making them productive and attractive for farmers, can help to change the game.

Our future climate-proof crops will require increased resilience to allow them to maintain their productivity in the face of the negative effects of climate change such as increased frequencies of extreme temperature, drought or salinity [14]. Importantly, future-proof crops will also be essential to mitigate the effects of climate change by enhancing below-ground carbon sequestration and contributing to improved soil health, resistance to erosion and fertility [15].

Given the increase in population, the pressure on land availability and the impacts of climate change, a sustainable increase in crop production cannot rely on further expansion of the agricultural area. Increasing the productivity of agriculture at no risk for finite natural resources will prevent additional unneeded land use for agriculture. While future yield increases will rely on substantially and sustainably increasing crop yields per hectare, in many countries and for many crops, further increases of crop yields are already constrained as agricultural practices are already very advanced, further land for agriculture is not available and two key crop yield related traits, the efficiency of light interception by canopy and the harvest index, are approaching their maximum value. These, as well as the fading out of pesticides and the reduction of fertilizer use, will increase the urgency to realign the breeding efforts in terms of goals and timely efficiency.

## 2. How?

Improving our crop varieties is one key action area for meeting the challenges as outlined. Crop breeding offers us the means for improving productivity, reducing nutrient and other chemical inputs, increasing efficiency of water use, promoting soil health; improving nutritional quality and ensuring that crops are resilient to the challenging conditions ahead.

Solar energy is plentiful, durable and accessible on a global scale as the Earth receives a staggering 162,000 TW of solar energy. To put this into perspective, one hour of the solar radiation intercepted by the Earth equals the total annual energy consumption of the entire global economy. The main challenge for the bio-society will be the capture and storage of this energy. Plants play a crucial role in this, as through photosynthesis, 2.8 ZJ (2.8 × 10^21^ joules) of solar energy is converted and stored as chemical energy annually. During this process, 451 gigatons of CO_2_ are fixed from the Earth’s atmosphere [16]. The key to achieving the future required global crop yield increases will be developing advanced crops with increased photosynthesis, which is the major yield-related plant trait that can still be substantially improved [17,18]. Currently, in temperate agricultural crops, the overall long-term efficiency of conversion of absorbed solar radiation to the energy content of biomass (ε_c_, a parameter in the Monteith model for crop productivity [17]) is approximately 0.5–1.3% [19,20,21] implying that for food production we miss around 99% of the available solar energy. Growth season estimates of ε_c_ are higher, at about one-third to one-half of the theoretical maximum efficiencies for solar energy conversion, which on a total solar irradiance basis are about 4.5% for C3 and 6% for C4 crops [22]. The difference between the achieved ε_c_ and the theoretical limits for ε_c_ implies that the scope for its improvement are considerable. Photosynthesis plays a major role in determining the value of ε_c_ so the opportunity is there to substantially increase the global crop yield by increasing the efficiency of plant photosynthesis [18,23]. The promise that crop yields could be increased by improving photosynthesis has been verified by several proof-of-principle experiments in which photosynthetic sub-traits were improved using genetic modification approaches [24,25,26]. These pioneering experiments showed that increasing photosynthesis by various routes results in increased plant biomass.

Redesigning our crop plants will not only imply the development of superior plants by optimizing plant photosynthetic efficiency for light. Plant productivity (and photosynthesis) may also be limited by the availability of other resources, such as water, or nutrients such as nitrogen or phosphorous. In many environments, these natural resources are, or are becoming, increasingly scarce [27]. Our future crop species should therefore also have an increased resource use efficiency. In addition, these plants should be equipped with an elevated abiotic stress resistance to cope with the already imminent negative effects of global climate change, such as increased temperature, drought, salinity and water stress, as well as with extreme events and anthropogenic pollutants.

Plant secondary metabolites that are also made by photosynthesis are underexplored and underused natural tools that may allow us to improve plant resistance and resilience to stresses [28]. In addition, those secondary metabolites not only protect plants, but are beneficial to humans as well, contributing to more diverse diets and human health. To this end, more diverse crops will contribute to food and nutritional security and to more resilient agricultural production. Traits targeting root architecture and function for better water, nitrogen, phosphorus or carbon use efficiency can also be exploited to save resources or enhance carbon in soils. By providing a higher capacity to capture atmospheric CO_2_, to improve source-sink relationships, and to better store carbon in woods and the soil, these plants will thus contribute to climate change mitigation and to improved soil health and fertility.

Finally, only now we begin to perceive the impact of microbiome of plants and soil as a main contributor of plant health and productivity [29]. This is also to be considered when working to climate-ready crops that are fully in balance with present and future ecosystems.

## 3. The CropBooster Program

In 2016, an initiative was launched by Wageningen University & Research with the working title “Photosynthesis 2.0”. The aim of the initiative was to explore the scientific options for increasing plant performance by increasing plant photosynthesis. This brought together a consortium of more than 60 universities and research institutes from 17 EU member states. In 2018, the consortium’s proposal to a Coordination and Support Action (CSA) was successful and the respective project to draft the roadmap for a future, large scale European research endeavor with the working title “The CropBooster Program” began. This CSA is aptly called “CropBooster-P” (H2020, GA 817690) where the “P” stands for “preparatory”, and the roadmap that the project is producing will be finalized in early 2022. The CropBooster-P roadmap basically resides on three pillars: scientific and technical possibilities to improve crop varieties, environmental, social and economic impact of the proposed improvements and societal acceptance.

In the first year, the project considered future scenarios [30] and undertook in-depth literature analyses of the current scientific state-of-the-art to improve crop yield and crop sustainability (defined as abiotic stress resistance and resource use efficiency). Included in these analyses were the plant biological options to improve plant nutritional quality for the human diet. Improving crop yield and sustainability should not be at the expense of nutritional quality, but contrarily, the envisaged high-yielding crops should even have an increased nutritional quality. The analyses focused on options to improve plants either by conventional breeding or by applying modern breeding technology. The results of these analyses are stored in a dedicated database [31] which currently holds approximately 900 keystone scientific publications, forming a comprehensive overview of the current options and possibilities to increase crop yield, nutritional quality and sustainability.

Complementing the literature survey, a modelling study was performed to assess the impact of increased photosynthetic efficiency on yield for a number of key crops in Europe. This study confirmed the potential of photosynthesis to drive significantly increased agricultural yields at different geographical locations in Europe.

The second year of the CropBooster-P project explored the social, environmental and economic impacts that the different identified scientific and technological options to improve crop varieties may hold, taking a stakeholder-led approach. Experts from farm-level through to businesses and supply chains to the consumer were consulted in a series of online workshops and surveys to prioritize the traits to breed for when future-proofing our crops, and to identify and discuss the impacts of adopting these technologies [32]. These were combined with rapid evidence reviews to give an indication of what is known about the wider impacts of crop breeding. Currently, the results of the impact analyses are being compiled and analyzed, and the results arising from this work will feed into the final roadmap the project is developing.

It is important that any future crop improvement program has the support of society at large. Ensuring food and nutritional security while at the same time mitigating the effects of global climate change and protecting Earth’s biodiversity might require a number of drastic measures, which will affect the life of everyone. Decisions will have to be made about the use of landscapes, business models for farming, prices of food and acceptance of novel technology to combine the advantages of all available approaches in crop improvement and management, just to mention a few. In order to ensure that decisions are made that have the support of major fractions of society, it is of imminent importance that (i) available options for crop improvement and consequences thereof are explained to the general audience and (ii) society is involved in decision making. For this, the CropBooster-P project is currently engaging with society (consumers and other non-expert laymen) in a series of workshops and events to research how complex scientific information can best be shared with the general public as well as different social actors and their perception and attitude towards aspects such as scientific research, plant breeding technology, food security, climate change and biodiversity. This is complemented by surveys among the public and stakeholders, which are already carried out and in progress at national level, for example in Norway, Finland and Switzerland [33,34,35].

The results of this research will lead to the drafting of a roadmap, ensuring that proposed technological options for future-proofing our crops will have maximal support from society at large.

The CropBooster-P roadmap will also contain a detailed research agenda, outlining the scientific program to be executed in the proposed CropBooster Program. This research agenda is developed in close concert with the European plant science community at large, and with the plant breeding sector. To do so, a detailed mapping of the European plant research landscape was performed identifying scientists, institutions and companies working on the topics of crop yield, nutritional quality and sustainability. Approximately 90 identified key scientists accepted an invitation to join one of 15 different focus groups, each directed at further deepening the science-base of a particular sub-topic related to increasing yield, quality and sustainability and were then created for structuring the European plant research landscape in these topics. The coordinators of the 15 focus groups, established contacts with an average of nine experts per focus group. Altogether, this approach involved more than 130 experts from 70 institutes or universities and 15 countries. The work of these focus groups was then discussed in a dedicated online conference [36], and the outcome of this will form the basis of the research agenda for the CropBooster Program.

The roadmap thus developed will outline the research agenda of the CropBooster Program, taking into account scientific knowledge, companies’ perspectives and societal views and concerns.

## 4. A Call for Action

The design and development of future-proof plants is bold, inspirational and a daunting task. They will be a true game-changer that will positively impact all levels of society and will instigate desirable, disruptive effects on all our ways of life. To succeed, we must unify virtually all sectors and all disciplines involved: farmers, distributors, processors and breeders in the agriculture sector; distributors, logistics experts, shipping and storage in the transport sector; the energy sector, regarding both energy supply as well as energy usage; the health sector, regarding aspects of food quality, food safety, nutrition and healthy diets; waste valorization and the bioeconomy; and, last but not least, supermarket and consumer organizations.

European plant scientists invite the research and innovation community in the agri-food chain to join in an interdisciplinary effort to organize the CropBooster Program, the successor to Cropbooster-P, as a pan-European research and innovation program aiming at producing our plants that support a sustainable future. The roadmap for the CropBooster Program is currently being drafted and will form the blueprint for both the research agenda to be executed, as well as the implementation route to introduce future-proofed crops.

We invite the European plant science community and all interested actors in the agri-food research and innovation to join the CropBooster Program. We also call upon the European Commission and Member States to support this initiative as one of the core instruments to achieve the ambitious targets of the European Green Deal, particularly its Farm to Fork and the Biodiversity strategies and related policies, to ensure food and nutritional security, preservation of natural resources and to combat global climate change.

## Data Availability

Not applicable.
